# Peripheral Inflammatory Cytokine Signature Mirrors Motor Deficits in Mucolipidosis IV

**DOI:** 10.3390/cells11030546

**Published:** 2022-02-04

**Authors:** Albert L. Misko, Laura D. Weinstock, Sitara B. Sankar, Amanda Furness, Yulia Grishchuk, Levi B. Wood

**Affiliations:** 1 Center for Genomic Medicine and Department of Neurology, Massachusetts General Hospital Research Institute, Harvard Medical School, 185 Cambridge St., Boston, MA 02114, USA; AMISKO@mgh.harvard.edu (A.L.M.); amandafurness26@gmail.com (A.F.); 2 Wallace H. Coulter Department of Biomedical Engineering at Georgia Tech and Emory, Georgia Institute of Technology, 315 Ferst Dr., Atlanta, GA 30332, USA; lweinstock3@gatech.edu (L.D.W.); sitarasankar2@gmail.com (S.B.S.); 3 George W. Woodruff School of Mechanical Engineering and Parker H. Petit Institute for Bioengineering and Bioscience, Georgia Institute of Technology, 315 Ferst Dr., Atlanta, GA 30332, USA

**Keywords:** lysosomal storage disorder, mucolipidosis, cytokines, blood, plasma, biomarkers, motor function

## Abstract

Background: Mucolipidosis IV (MLIV) is an autosomal recessive pediatric disease that leads to motor and cognitive deficits and loss of vision. It is caused by a loss of function of the lysosomal channel transient receptor potential mucolipin-1 and is associated with an early pro-inflammatory brain phenotype, including increased cytokine expression. The goal of the current study was to determine whether blood cytokines are linked to motor dysfunction in patients with MLIV and reflect brain inflammatory changes observed in an MLIV mouse model. Methods: To determine the relationship between blood cytokines and motor function, we collected plasma from MLIV patients and parental controls concomitantly with assessment of motor function using the Brief Assessment of Motor Function and Modified Ashworth scales. We then compared these profiles with cytokine profiles in brain and plasma samples collected from the *Mcoln1^−/−^* mouse model of MLIV. Results: We found that MLIV patients had prominently increased cytokine levels compared to familial controls and identified profiles of cytokines correlated with motor dysfunction, including IFN-γ, IFN-α2, and IP-10. We found that IP-10 was a key differentiating factor separating MLIV cases from controls based on data from human plasma, mouse plasma, and mouse brain. Conclusions: Our data indicate that MLIV is characterized by increased blood cytokines, which are strongly related to underlying neurological and functional deficits in MLIV patients. Moreover, our data identify the interferon pro-inflammatory axis in both human and mouse signatures, suggesting that interferon signaling is an important aspect of MLIV pathology.

## 1. Introduction

Mucolipidosis type IV (MLIV) is a neurodevelopmental and neurodegenerative disorder caused by the loss of function of mucolipin 1 (TRPML1), a lysosomal channel encoded by the *MCOLN1* gene. Patients typically present with delayed developmental milestones in the first year of life and reach a plateau in psychomotor function equivalent to the 18-to-20-month range [1]. Across their lifespan, patients exhibit progressive visual impairment due to retinal degeneration and corneal clouding, leading to blindness, achlorhydria, worsening muscular hypertonicity, and deteriorating motor function [1,2]. All of these features are recapitulated in *Mcoln1* knockout mice (*Mcoln1*^−/−^, KO) [3,4,5]. Atypical MLIV patients with milder neurological impairment have also been recognized, and the attenuated disease severity is attributed to residual TRPML1 function demonstrated with some allelic variants.

MLIV results in a hypomyelinating leukodystrophy with iron accumulation in the basal ganglia and progressive cerebellar atrophy. Brain imaging in MLIV patients and histopathological evaluation of *Mcoln1*^−/−^ mice demonstrate a paucity of subcortical white matter, hypoplasia/dysgenesis of the corpus callosum, and variable white matter lesions [6]. In patients, subcortical white matter and cerebellar volumes decrease with age, while cortical gray matter volumes are relatively preserved [7]. In parallel, pathological abnormalities in *Mcoln1*^−/−^ mice primarily manifest in the form of reduced myelination, astrocytosis, microgliosis, and partial loss of cerebellar Purkinje cells, while cortical neuron populations are largely unaffected [3,8,9].

We previously found that signs of neuroinflammation, including astrocytosis, microgliosis, and increased expression of numerous pro-inflammatory cytokines/chemokines, are present in the brains of *Mcoln1*^−/−^ mice early in the course of disease, before the functional symptoms first emerge [10]. Because a growing body of data suggests that cytokine/chemokine signaling may affect the maturation and function of oligodendrocytes and Purkinje cells [11,12] and drive chronic neurodegeneration [13], we reasoned that cytokine/chemokine signaling may play a role in the pathogenesis of MLIV. Additionally, our prior work identified a marked increase in interferon gamma-inducible protein 10 (IP-10) in both whole brain cortical tissues and isolated astrocytes from *Mcoln1*^−/−^ mice, suggesting that the interferon pathway may be strongly linked to disease progression in MLIV.

Because interferons are known to pass through the blood–brain barrier, we set out to identify cytokine/chemokine signatures in plasma from MLIV patients and *Mcoln1*^−/−^ mice. We hypothesized that interferon levels would be strongly linked to motor dysfunction in human MLIV patients. To test this, we collected blood plasma from MLIV patients and familial controls while simultaneously measuring gross and fine motor scores in MLIV patients. By quantifying 41 cytokines/chemokines in the plasma, we found that cytokines were broadly upregulated in MLIV patients compared to familial heterozygous controls. Moreover, among MLIV patients, those with reduced fine or gross motor function had robustly increased blood cytokines, including TNF-α, IFN-γ, and IFN-α2. To test whether these findings would translate to mice, we also analyzed 32 cytokines in blood plasma from *Mcoln1*^−/−^ mice at one, two, and six months of age, corresponding to the presymptomatic, early-symptomatic, and late stages of the disease in mice. Our analysis revealed a robust proinflammatory cytokine profile that changed with age, including trending or significantly increased IP-10 at all time points. Finally, we found that several blood cytokines from human or mouse plasma overlapped with cytokines expressed in the MLIV mouse brain, including IP-10, suggesting that these cytokines may be candidate biomarkers of brain pathology in MLIV. In total, our findings reveal that MLIV blood cytokine signatures are strongly associated with the severity of motor dysfunction in patients and suggest that peripheral cytokine signature may reflect the brain’s neuroinflammatory milieu.

## 2. Materials and Methods

### 2.1. Study Design and Population

Patients with MLIV (*N* = 18, F = 9 and M = 9) and familial controls (*N* = 19, F = 11 and M = 8) were recruited through the Mucolipidosis Type IV Foundation. Written informed consent was obtained from legal guardians for participation in our approved natural history and biomarkers studies according to protocols approved by the Massachusetts General Hospital Institutional Review Board. Written informed consent or assent were obtained from patients if their neurological capacity allowed. Inclusion criteria for patients included a documented diagnosis of MLIV by (1) clinical or research-based sequencing of *MCOLN1* and identification of two pathological *MCOLN1* alleles or (2) presence of the expected constellation of clinical symptoms associated with MLIV and documentation of at least one of the following: one pathological *MCOLN1* allele, elevated gastrin levels, or a tissue biopsy with evidence of lysosomal inclusions consistent with MLIV. One familial control was excluded from cytokine analysis when it was later revealed that this subject had undergone a surgical procedure in the week prior to sample collection, which could affect cytokine levels. MLIV cases were assigned to either typical (*N* = 15) or mild (*N* = 3) presentation. Based on our clinical experience, we classified patients as mild if they had attained independent ambulation at some point in life. All patients underwent a full examination by a single board-certified pediatric neurologist, and their function was scored with the Brief Assessment of Motor Function (BAMF) scales (gross motor, upper extremity gross motor, fine motor, deglutition and articulation) and Modified Ashworth scale. Due to limitations associated with patient access and cost, repeat scoring by independent evaluators was not possible.

### 2.2. Blood Sample Collection and Processing

Blood was collected in K_2_EDTA-coated Purple/Lavender top vacutainers (BD 368047). Plasma was isolated within 4 h from collected blood samples by centrifugation at 1000 g for 10 min at 4 °C. Isolated plasma was stored at −80 °C.

### 2.3. Animal Studies and Sample Collection

*Mcoln1*^−/−^ knockout (KO) mice (on a C57Bl/6J background) were maintained and genotyped as previously described [3,8,9]. The study was performed in a blinded manner to avoid bias. Mice were identified using four-digit IDs on their ear tags, and the study staff were blinded to mouse genotypes at the time of sample collection. After genotyping, mice were randomly placed in cages by 3 or 4 mice per cage to form the experimental cohorts. Sample sizes have been selected based on our previous study [10], and no mice were excluded from the experimental cohorts at the time of sample collection. Blood was collected from *Mcoln1^−/−^* (KO) and littermate *Mcoln1^+/+^* (WT) controls at 1 (*N* = 6 WT, 8 KO males, 6 WT, 8 KO females), 2 (*N* = 6 WT, 8 KO males, 4 WT, 8 KO females), and 6 (*N* = 6 WT, 6 KO males, 8 WT, 8 KO females) months of age, and plasma was isolated within 4 h from collected blood samples by centrifugation at 1000*g* for 10 min at 4 °C. Isolated plasma was stored at −80 °C. All experiments were performed according to the U.S. National Institutes of Health guidelines and approved by the Massachusetts General Hospital Institutional Animal Care and Use Committee.

### 2.4. Cytokine Luminex Immunoassays

Plasma was stored at −80 °C. For human cytokine analysis, samples were diluted to 8% in Milliplex assay buffer and analyzed using the Milliplex MAP Human Cytokine/Chemokine Magnetic Bead Panel—Premixed 41 Plex kit (Millipore Sigma, St. Louis, MO, USA, HCYTMAG-60K-PX41). Mouse plasma was diluted to 70% in assay buffer and analyzed using the Milliplex MAP Mouse Cytokine/Chemokine Multiplex assay (Millipore Sigma, St. Louis, MO, USA, MCYTMAG-70K-PX32). Assays were read out with a MAGPIX Luminex instrument (Luminex, Austin, TX, USA).

### 2.5. Statistical and Multivariate Analyses

Dunn’s test was performed in R (R Foundation for Statistical Computing, Vienna, Austria) and adjusted using a Bonferroni correction for multiple comparisons. Wilcoxon rank sum test was performed in R. Partial least squares regressions (PLSRs) and discriminant PLSRs (D-PLSRs) were performed in R using the ropls package v1.4.2. The data were z-scored before input into the function. Cytokine measurements were used as the independent variables, and the discrete regression variable in all D-PLSR analyses was genotype/phenotype. Orthogonal rotations were applied to the sample scores and analyte weightings to obtain consistent separation of each group along the LV1 and LV2 axes. Error bars for LV loadings were calculated by iteratively excluding K samples without replacement 100 times (leave-K-out cross-validation, LKOCV), and regenerating the D-PLSR model each time. Error bars in the LV1 plots report the mean and SD computed across the models generated to provide an indication of the variability within each cytokine among the models generated. Sample outliers were checked for and removed in the mouse cytokine data by performing a principal component analysis on the data and iteratively removing data points that fell outside of a 99.5% confidence ellipse (mahalanobisQC in ClassDiscovery package v3.3.13).

## 3. Results

### 3.1. Mucolipidosis Type IV Patients Exhibit Pro-Inflammatory Blood Signatures Compared to Familial Controls

**Figure 1:**
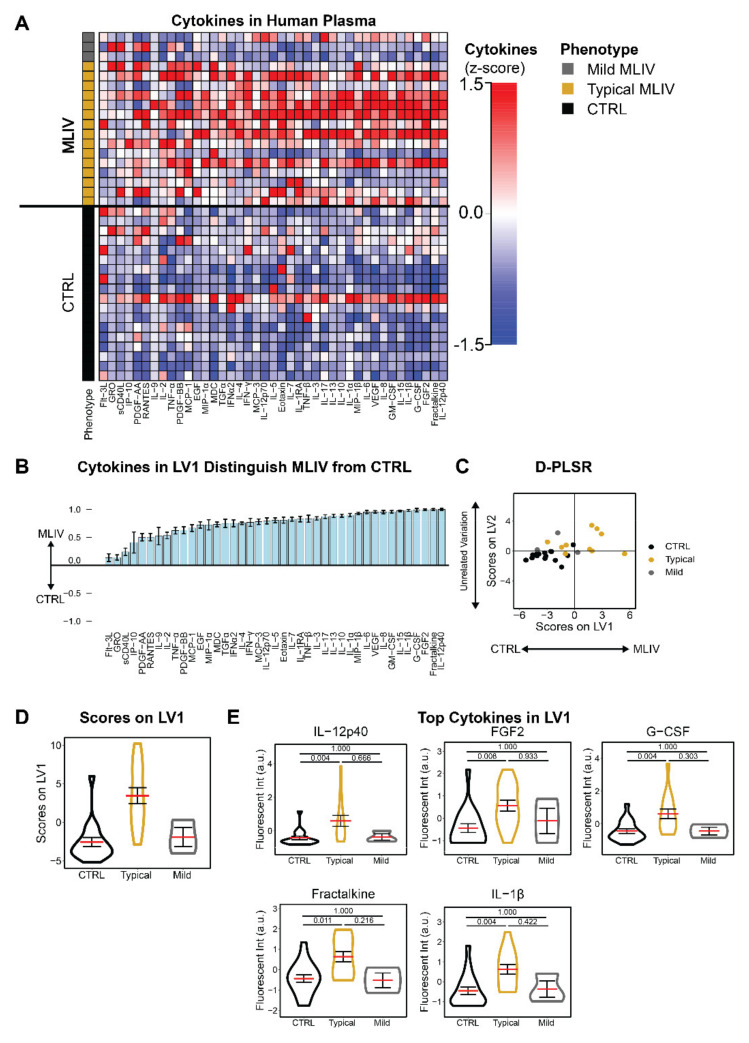
Plasma cytokines are increased in MLIV patients compared to familial controls. (**A**) Panel of 41 cytokines measured from plasma samples using a multiplexed immunoassay. Each column is z-scored, *N* = 18 CTRL, 18 MLIV (15 typical and 3 mild). (**B**) A discriminant partial least squares regression (D-PLSR) was used to simultaneously regress all measured cytokines against disease group (CTRL, MLIV). The D-PLSR identified a profile of cytokines, called a latent variable 1 (LV1), that separated MLIV cases (positive) from CTRL cases (negative) (mean ± SD in a leave-K-out cross-validation (LKOCV) with K = 5). (**C**) LV1 separated MLIV cases to the right and both CTRL and mild cases to the left. **(D)** Violin plots showing scoring of samples in (**C**) in each group (mean ± SEM) along LV1. *N* = 18 CTRL, 18 MLIV (15 typical and 3 mild). (**E**) Violin plots showing univariate analysis of top cytokines from LV1 (panel B, data are represented as mean ± SEM). Statistical testing was conducted via Dunn’s test with Bonferroni correction, and associated significance values are annotated for each comparison at the top of each plot). *N* = 18 CTRL, 18 MLIV (15 typical and 3 mild).

Having previously identified robust neuroinflammatory changes in the CNS of *Mcoln1*^−/−^ mice [10], here, we aimed to identify a cytokine signature associated with MLIV in patient plasma compared to familial controls. We collected samples from 18 MLIV patients, including three mild cases (Table 1), and 18 parental controls and used a Luminex multiplexed immunoassay to simultaneously quantify 41 cytokines from each sample. Our analysis revealed striking differences in blood cytokine levels in MLIV patients compared with controls (Figure 1A). To account for the multidimensional nature of the data, we used a discriminant partial least squares regression (D-PLSR) to identify cytokines that best distinguished MLIV patient samples from controls [10]. The D-PLSR analysis identified a weighted profile of cytokines, called a latent variable (LV1), that best distinguished MLIV samples from controls (Figure 1B). Error bars representing the mean ± SD were generated by iteratively leaving K = 5 samples out and regenerating the D-PLSR model, and they indicate that the model is not disproportionately influenced by a small number of samples (leave-K-out cross-validation, LKOCV). Scoring all the samples (Figure 1A) based on the cytokine profile in LV1 separated control samples to the left and MLIV samples to the right (Figure 1C). Importantly, the analysis found mild cases to group together with control cases, suggesting that the cytokine signature on LV1 segregates samples based on clinical severity (Figure 1D). Univariate analysis of the top five cytokines from LV1 revealed significant individual differences between typical MLIV and familial control patient samples (Figure 1E) but not between mild and control samples (univariate statistical analysis of all 41 measured cytokines is given in Table S1).

### 3.2. Plasma Cytokines Are Increased in Patients with Poor Motor Function and Hypertonicity

**Figure 2:**
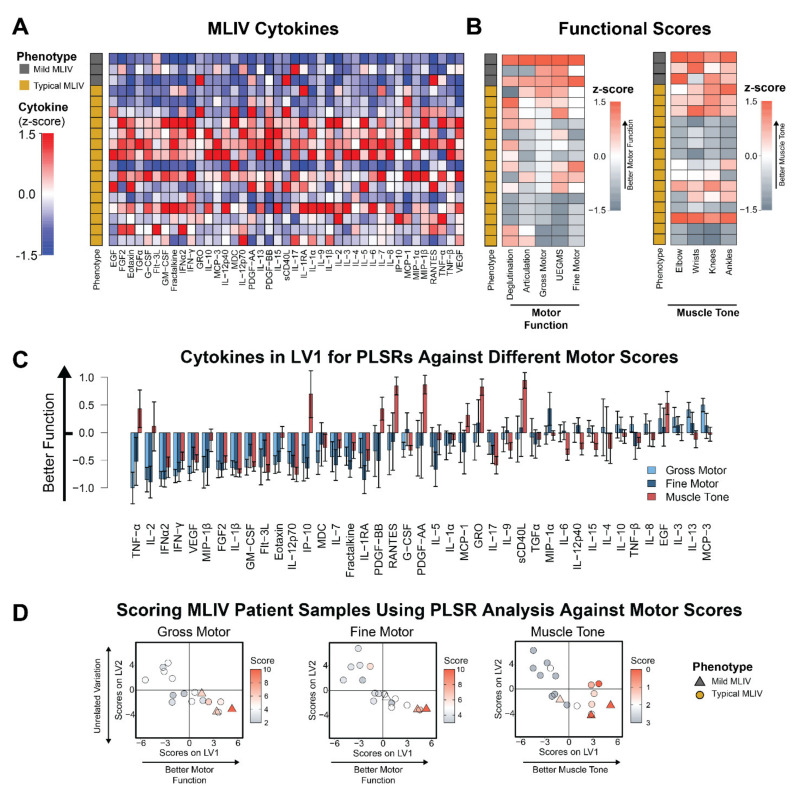
Blood cytokine signatures are related to motor dysfunction in MLIV patients. (**A**) Heatmap of relative cytokine expression for MLIV samples (columns are z-scored). *N* = 18 MLIV (15 typical and 3 mild). (**B**) Brief Assessment of Motor Function (BAMF) and Modified Ashworth scores for all MLIV patients (z-scored). Rows are sorted to match cytokine samples in panel A. (**C**) PLSR regressions of MLIV patient cytokines against gross motor, fine motor, and muscle tone (elbow) reveal profiles of cytokines (LV1) that correlate with poorer function (negative) or better function (positive) (mean ± SD in a LKOCV with K = 3). (**D**) Scoring each MLIV patient sample based on the overall cytokine expression profile for each sample and each regression separated samples with poorer function to the left and better function to the right along LV1. The second axis, LV2, was associated with unrelated variation. Mild cases are shown as triangles and typical cases are shown as circles.

Given the group-wise differences between typical and mild MLIV patients, we next asked if plasma cytokine levels were related to clinical disease severity. To test this, we performed an analysis of plasma cytokine levels and motor function scores for each patient. We used the Brief Assessment of Motor Function (BAMF) [14,15,16,17] and the Modified Ashworth scale [18] to score domains of motor function and muscle tone, respectively, in all MLIV patients simultaneously with an analysis of plasma cytokines (Figure 2A,B).

To identify the cytokines that were most strongly associated with the BAMF and Modified Ashworth scores, we next used partial least squares regression (PLSR) analysis to separate patients with better motor scores to the right and with reduced motor scores to the left (Figure 2C,D). Interestingly, we found that reduced gross motor and fine motor function strongly correlated with pro-inflammatory cytokines, including IL-2, TNF-α, MIP-1β, IFN-γ, and IFN-α2 (Figure 2C). The PLSR analysis also revealed a profile of cytokines strongly associated with increased (i.e., worse) muscle tone. The top cytokines associated with increased muscle tone include IL-12p70, IL-17, IFN-α2, and IL-1β. The limited overlap between cytokines correlating with lower gross/fine motor function and increased (i.e., worse) muscle tone may suggest that these disease features are linked to different inflammatory processes. Importantly, we also used PLSR to correlate cytokines against both age and sex and found that among the top five correlates of each, only TNF-α and IL-2 overlapped with the top five correlates from the gross motor, fine motor, and muscle tone analyses (Figure S1). These findings indicate that the top cytokines associated with motor function or tone are only partially driven by sex and age.

### 3.3. Plasma Cytokines Are Increased in Mcoln1^−/−^ Mice

**Figure 3:**
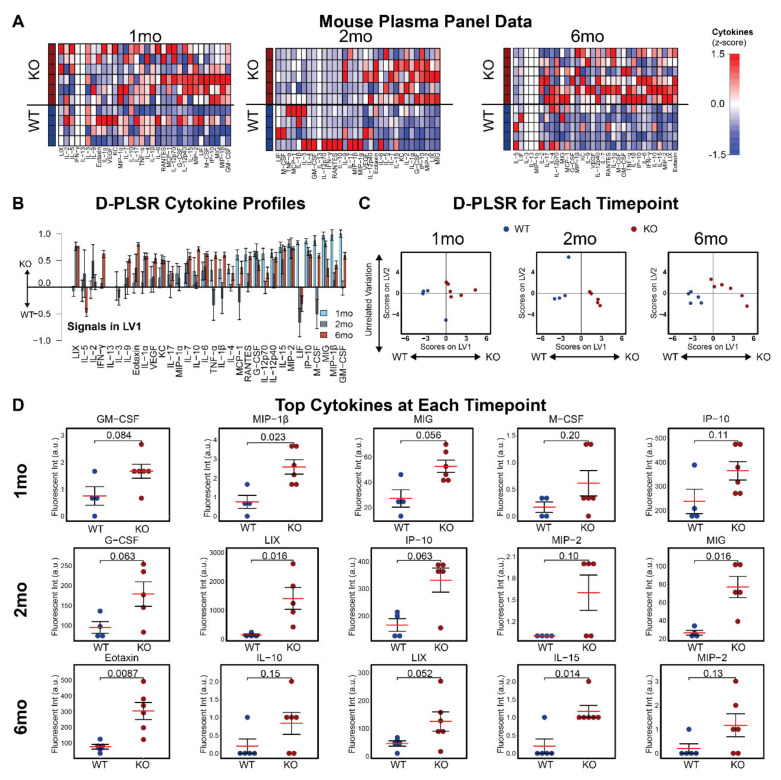
Plasma cytokine signatures distinguish male *Mcoln1*^−/−^ mice from wild-type controls. (**A**) A panel of 32 cytokines quantified from blood plasma in WT and *Mcoln1*^−/−^ (KO) animals at 1 (*N* = 4 WT, 6 KO), 2 (*N* = 4 WT, 5 KO), and 6 (*N* = 5 WT, 6 KO) months of age using a multiplexed immunoassay (each column is z-scored). (**B**) D-PLSR analysis at each timepoint reveals cytokine signatures associated with WT (negative) or KO (positive) mice (mean ± SD in a LKOCV with K = 1). Cytokines are ranked such that those most associated with 1 mo KO mice are shown on the right, and those most associated with 1 mo WT mice (negative) are shown on the left. (**C**) Scoring each sample based on its own LV1 profile in (**B**) separates WT mice to the left and KO mice to the right at each time point. (**D**) Univariate analysis of top cytokines from LV1 at 1 (*N* = 4 WT, 6 KO), 2 (*N* = 4 WT, 5 KO), and 6 (*N* = 5 WT, 6 KO) months of age (data shown as mean ± SEM; statistical testing was performed with Wilcoxon rank sum test, and associated significance values are annotated at the top of each plot).

Having found strong relationships between cytokine profiles and motor function in human subjects, we next asked if *Mcoln*^−/−^ mice exhibit a similar relationship between plasma cytokines and progression of disease. Identification of a relationship between cytokine levels and disease state in mice will enable the use of cytokines as biomarkers for pre-clinical testing. To test this relationship, we collected plasma from mice at 1, 2, and 6 months of age in male (Figure 3A) and female (Figure S2A) mice. We used D-PLSR analysis to identify profiles of cytokines that distinguished *Mcoln1*^−/−^ from wild-type controls (Figure 3B and Figure S3B). At all time points in males, *Mcoln1*^−/−^-associated cytokines in each profile included GM-CSF, MIP-1β, MIG, and IP-10, all of which have pro-inflammatory or chemotactic properties (Figure 3B–D, Table S2) [19,20]. Female *Mcoln*^−/−^ mice showed a similar trend, with top correlates at 1 month of age including IL-12p70, IP-10, IL-15, and MIG, all of which are chemotactic or pro-inflammatory (Figure S2, Table S3) [20,21,22]. Interestingly, the pro-inflammatory cytokine profile was subdued in 6-month-old female mice compared to controls (Figure S2B–D, Table S3). Together, these data demonstrate that *Mcoln1*^−/−^ mice exhibit a strong pro-inflammatory plasma signature. Moreover, several of these cytokines, including IP-10, overlap with plasma correlates of poor motor function in MLIV patients (Figure 2C), highlighting the strength of the *Mcoln1*^−/−^ model of MLIV for pre-clinical research.

### 3.4. Plasma Cytokine Signatures Overlap with Brain Cytokine Signature in Mcoln1^−/−^ Mice

**Figure 4:**
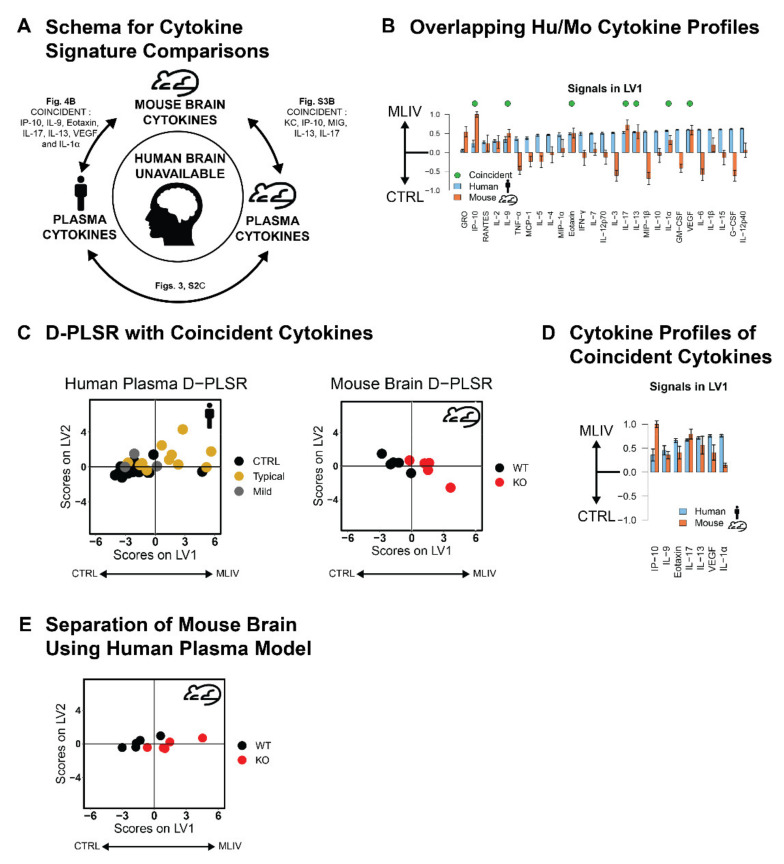
Coincident cytokine signature distinguishes human MLIV plasma and mouse brain samples. (**A**) Concept for identifying potential cytokines that may predict human MLIV brain condition based on cytokines measured in the human and mouse plasma, as well as on cytokine overlap between mouse brain and plasma. (**B**) LV1 cytokine profiles distinguishing mouse brain and human plasma generated based on 26 overlapping cytokines in human and mouse datasets. Of these, 7 cytokines were coincident in directionality to separate human plasma and mouse brain MLIV samples from controls (mean ± SD in a LKOCV with K = 3 for human samples, K = 1 for mouse). (**C**) D-PLSR analysis with 7 coincident cytokines separated both human plasma and mouse brain samples from controls along LV1, suggesting that this reduced cytokine signature is capable of separating control and MLIV samples from both human plasma and mouse brain samples. *N* = 18 CTRL, 18 MLIV (15 typical and 3 mild) for human samples and *N* = 5 WT, 5 *Mcoln1*^−/−^ for mouse samples. (**D**) Reduced 7 cytokine LV1 profiles distinguishing MLIV and control cases for human plasma and mouse brain. (**E**) D-PLSR model generated based on 7 cytokines from human plasma separated wild-type mice to the left and KO mice to the right based on brain cytokine measurements.

A key challenge in interpreting blood cytokine signatures in MLIV patients is determining their relevance to neuroinflammation and brain pathology, given that cytokines may be sourced from multiple cell types and compartments throughout the body. Although it is impossible to fully distinguish the sources of cytokines found in the plasma, we next asked if differences in cytokines measured in the plasma from MLIV patients would reflect cytokine differences previously found in the MLIV brain. Since postmortem brain tissues have only been collected from one MLIV patient [23], we instead took advantage of our previously published cytokine data quantified in the cerebral cortices of 2-month-old female *Mcoln1*^−/−^ and wild-type mice [10] to build D-PLSR models that could be used to compare our human and mouse plasma signatures (Figure 4A) presented in Figure 1, Figure 2 and Figure 3. We first identified coincident cytokines that commonly separated MLIV patients from controls and *Mcoln1*^−/−^ (KO) from wild-type mice based on the LV1 profiles, including the 26 cytokines that overlapped between the human and mouse panels. The coincident cytokines, IP-10, IL-9, Eotaxin, IL-17, IL-13, VEGF, and IL-1α, were selected based on having mean values on LV1 (≥0.2) and coefficients of variation (<1) (Figure 4B). This resulted in a reduced set of seven cytokines coincident between human plasma and mouse brain that distinguished MLIV from control samples in both human plasma and mouse brain (Figure 4D). Additionally, when we used the coincident D-PLSR human plasma cytokine signature (Figure 4C) to analyze mouse brain data [10], we found that the human plasma signature separated *Mcoln1*^−/−^ mouse brain samples to the right and control samples to the left (Figure 4E). We conducted a similar analysis using the mouse plasma data to separate the mouse brain samples (Figure S3). This analysis revealed five coincident cytokines between mouse brain and mouse plasma, KC, IP-10, MIG, IL-13, and IL-17, which were similarly able to separate wild-type from *Mcoln1*^−/−^ mouse brain samples. We noted that only IP-10 was shared between each of these coincident cytokine signatures and that it was both significantly elevated in typical human MLIV cases (*p* < 0.001, Table S1) and either significantly or trending elevated in male or female Mcoln1^−/−^ KO mice at all time points (male 1 mo: *p* = 0.114, male 2 mo: *p* = 0.063, male 6 mo: 0.082, female 1 mo: 0.002, female *p* = 0.028, female *p* = 0.101, Tables S2 and S3). Given that IP-10 is a strong indicator of MLIV across human plasma, mouse plasma, and mouse brain, it is a particularly strong candidate to be considered in future work as a potential predictive biomarker.

## 4. Discussion

In this study, we extended upon our previous finding of disrupted cytokine homeostasis in the *Mcoln1*^−/−^ mouse brain and demonstrated profoundly altered peripheral cytokine levels in the plasma of *Mcoln1*^−/−^ mice and MLIV patients. Based on previous evidence of neuroinflammation in the brains of early symptomatic *Mcoln1*^−/−^ mice and the ability of cytokines to pass through the blood–brain barrier, we aimed here to establish peripheral cytokine profiles in MLIV patients and *Mcoln1*^−/−^ mice and to test whether peripheral cytokines correlate with disease progression. Indeed, our data showed that a profile of cytokines in blood plasma correlated with a loss of gross and fine motor function as well as increased muscle tone. Further, we found an overlap between cytokine signatures in plasma collected from MLIV patients and *Mcoln1*^−/−^ mice and between plasma signatures and the *Mcoln1*^−/−^ mouse brain. Taken together, our data suggest that plasma cytokine profiles reflect neuroinflammation in the CNS of MLIV patients. Further understanding of the roles of these cytokine alterations in disease pathogenesis may provide an accessible means of monitoring disease progression or suggest new targets for therapeutic intervention.

We identified a pro-inflammatory cytokine signature in the plasma of MLIV patients that correlated with impaired gross and fine motor function and increased muscle tone assessed via Brief Assessment of Motor Function (BAMF) and Modified Ashworth scales. These signatures included elevations of IL-2, TNF-α, MIP-1β, IFN-γ, and IFN-α2 (Figure 2C), which were associated with reduced gross and fine motor function, and IL-12p70, IL-17, IFN-α2, and IL-1β, which were the top cytokines associated with increased (worse) muscle tone. We have also identified cytokine signatures for different stages of disease progression in *Mcoln1*^−/−^ mice, including presymptomatic (1 month of age), early-symptomatic (2 months) and late (6 months) time points, and identified the top dysregulated cytokines for each of them (Figure 3 and Figure S2). We did not assess motor function in the mice used for the current study, so it was not possible to directly correlate cytokine levels with individual motor function impairment. Nevertheless, motor function decline has been thoroughly established by us and others in this model. In particular, *Mcoln1*^−/−^ mice appear healthy at birth and indistinguishable from wild-type and heterozygous littermates during the first two months of life. The first sign of motor function decline can be detected in the form of reduced vertical activity in the open field test at 2 months of age, after which vertical activity continually declines with age [24]. Starting at 4 months of age, *Mcoln1*^−/−^ mice develop balance and coordination deficits, resulting in shorter rod retention time in the rotarod test [24] and worsening performance in the balance beam test [25]. Motor dysfunction progresses further in aging mice, resulting in significant gait deficits and hind limb paralysis, requiring euthanasia by 6–8 months of age [24,26]. Therefore, while direct analysis of plasma cytokines and motor function in individual mice is not presented here, this detailed description of gross motor function decline in *Mcoln1*^−/−^ mice enables us to match dysregulated cytokines at three time points of disease progression with various levels of motor dysfunction. Plasma cytokines associated with worse motor scores in MLIV patients (TNF-α, IFN-α2, IFN-γ, and IP-10 (Figure 2C)) have been implicated in microglial activation, demyelination, and neuronal death [27,28]. It is important to note, however, that cytokines can play both neuroprotective and neurodegenerative roles. The relationship between altered cytokine levels and their impact on the CNS is complex and depends on the concentration, the specific effects of individual cytokines, the global neuroinflammatory state, and the developmental context; therefore, we cannot present firm conclusions about the mechanistic role of elevated cytokines in the pathogenesis of MLIV. However, the results of our current study lay the essential groundwork for future investigations, which could target the cytokines/chemokines identified here and test their impact on developmental myelination and/or degeneration of the subcortical white matter tracts and cerebellum.

TRPML1 has a well-established role in regulating a broad range of lysosomal functions [29,30], and recent data suggest its role in immune cell function [31,32,33,34,35]. TRPML1 activity promotes nuclear translocation of transcriptional factor EB (TFEB) [36], resulting in the activation of the CLEAR network of genes, which include lysosomal hydrolases, genes of lysosomal biogenesis, and autophagy [37,38]. Interestingly, transcription factors EB and E3 have also been linked to the transcriptional regulation of innate and adaptive immunity, including the transcription of CCL5, TNF-α, and IL-1β or IL-6 [39]. Therefore, the TRPML1–TFEB regulatory loop may play a central role in the transcriptional regulation of immune cells. TRPML1 has also been shown to play a role in macrophage phagophore formation, the migration of dendritic cells, and the regulation of the effector activity of natural killer cells [31,32,33]. Additionally, loss of TRPML1 leads to pro-inflammatory activation of microglia and a disease-associated transcriptomic signature in MLIV mouse microglia [40], although the functional consequences of these changes and the role of microglia in the pathophysiology of MLIV are still not fully understood.

Though several studies demonstrate the role of TRPML1 in regulating immune cell functions in in vitro systems [31,32,33,35], no peripheral immune-related clinical manifestations have yet been documented in MLIV patients. The reason for the lack of immune symptoms can be explained by compensatory mechanisms in the immune system, potentially via an TRPML1 ortholog, TRPML2. TRPML1 and TRPML2 share structural homology and can functionally substitute for each other, as demonstrated in ex vivo studies [41,42]. Perhaps the most striking difference between the two channels is their tissue specificity. Unlike TRPML1, which is ubiquitously expressed at a stable level in all examined tissues and cell types, the expression of TRPML2 is limited to lymphoid tissue, particularly thymus, spleen, and immune cells [43,44]. Therefore, it is tempting to speculate that the expression of TRPML2 in this selected set of tissues may be able to prevent gross consequences of TRPML1 loss of function and spare some aspects of immune function in patients with MLIV. This is supported by data showing that TRPML1-deficient B-lymphocytes expressing TRPML2 do not manifest lysosomal pathology typically observed in TRPML1-deficient cell types (skin fibroblasts, neural cells, etc.) that do not endogenously express TRPML2 [45]. The role of lysosomes in mediating multiple aspects of immune cell function is well established [46,47,48], and signs of neuroinflammation, including altered cytokine levels, have been reported in several lysosomal storage diseases (LSD), including Gaucher disease, Fabry disease, Niemann–Pick disease, and MPS [49,50,51,52]. However, surprisingly little is known about systemic immune dysfunction in other LSDs, and a systematic approach to establish peripheral signature of cytokines/chemokines for LSDs is lacking. Here we show that blood cytokines can be linked to the severity of CNS dysfunction in the lysosomal disease mucolipidosis IV, which offers new possibilities to track disease progression in MLIV patients and pre-clinical models.

Overexpression of cytokines, including interferons, in the brain during neurodegenerative process has led to studies determining whether interferons and other cytokines can pass through the blood–brain barrier, as well as whether cytokines may serve as a blood biomarker of neuroinflammatory dysfunction or neurodegeneration, e.g., in Alzheimer’s disease [53].

## 5. Conclusions

Collectively, our mouse and human data suggest an intricate relationship among neuroinflammation, peripheral cytokines, and loss of motor function in MLIV. Although our data do not reveal whether the commonalities between the brain and periphery are due to cytokines crossing the blood–brain barrier or due to a common mechanism between the brain and peripheral compartments, they do reveal that the peripheral cytokine signature is strongly associated with motor dysfunction in MLIV. Given this robust relationship and the likelihood that new therapies will simultaneously target the brain and peripheral compartments, cytokines hold strong potential to serve as an early indication of patient response to forthcoming therapeutic strategies for this devastating disease.

## Figures and Tables

**Table 1:** Clinical and genotype data for study subjects.

Patient	Age (Years)	Mode of Diagnosis	Phenotype	*MCOLN1* Genotype	
1	<5	Genetic	Typical	c.304 C > T	c.1405A > G
2	<5	Genetic	Typical	c.406 − 2A > G	c.1336G > A
3	<5	Genetic	Typical	c.1017_1020delACGG	c.877 G > A
4	5–10	Genetic	Typical	c.984 + 1G > A	c.406 − 2A > G
5	5–10	Genetic	Typical	c.406 − 2A > G	c.964C > T
6	5–10	Genetic	Typical	c.694A > C	c.785T > C
7	11–15	Genetic	Typical	c.406 − 2A > G	c.406 − 2A > G
8	11–15	Genetic	Mild	c.406 − 2A > G	c.406 − 2A > G
9	11–15	Genetic	Typical	c.406 − 2A > G	c.406 − 2A > G
10	11–15	Genetic	Typical	c.406 − 2A > G	c.406 − 2A > G
11	11–15	Genetic and Corneal bx	Typical	c.406 − 2A > G	Not Identified
12	16–20	Genetic	Typical	c.236_237ins93	c.694A>C
13	16–20	Genetic	Typical	c.406 − 2A > G	c.406 − 2A > G
14	16–20	Genetic	Mild	c.236_237ins93	c.1207C > T
15	25–30	Clinical and Gastrin	Typical	Unknown	Unknown
16	25–30	Genetic	Typical	c.406 − 2A > G	c.406 − 2A > G
17	25–30	Genetic	Typical	c406 − 2A > G	c406 − 2A > G
18	25–30	Genetic	Mild	c.920delT	c.1615delG

## Data Availability

All de-identified data tables used herein are available upon request.

## Supplementary Materials

The following are available online at https://www.mdpi.com/article/10.3390/cells11030546/s1, Figure S1: Blood cytokine signatures are related to sex and age in MLIV patients, Figure S2: Plasma cytokine signatures distinguish female *Mcoln1*^−/−^ MLIV mice from wild-type controls, Figure S3: Coincident cytokine signature distinguishes mouse MLIV plasma and mouse brain samples, Table S1: Statistical analysis of group-wise cytokines differences between CTRL, mild, typical cases, Table S2: Statistical analysis of group-wise cytokines differences between male WT and KO mice, Table S3: Statistical analysis of group-wise cytokines differences between female WT and KO mice.
