# Supplementary material for: Peripheral Inflammatory Cytokine Signature Mirrors Motor Deficits in Mucolipidosis IV
Source: Cells. 2022 Feb 4;11(3):546. doi: 10.3390/cells11030546 (PMC8834097; doi:10.3390/cells11030546)
Supplement: Supplementary file 1 [file cells-11-00546-s001.zip › cells-1498772-supplementary.pdf]

Peripheral Inflammatory Cytokine Signature Mirrors Motor Deficits in Mucolipidosis IV

Albert L. Misko[1,#], Laura D. Weinstock[2,#], Sitara B. Sankar[2], Amanda Furness[1], Yulia Grishchuk[1,¥,*], Levi B. Wood[2,3,¥,*]

* Correspondence should be addressed to Levi B Wood (levi.wood@me.gatech.edu) and Yulia Grishchuk (ygrishchuk@partners.org).

¥Equally contributing senior authors

[#]Equally contributing first authors

1- Center for Genomic Medicine and Department of Neurology, Massachusetts General Hospital Research Institute, Harvard Medical School, 185 Cambridge St., 02114 Boston, Massachusetts, USA;

2- Wallace H. Coulter Department of Biomedical Engineering at Georgia Tech and Emory, Georgia Institute of Technology, 315 Ferst Dr., 30332 Atlanta, Georgia, USA;

3- George W. Woodruff School of Mechanical Engineering and Parker H. Petit Institute for Bioengineering and Bioscience, 315 Ferst Dr., 30332 Atlanta, Georgia, USA;

Supplementary Figures

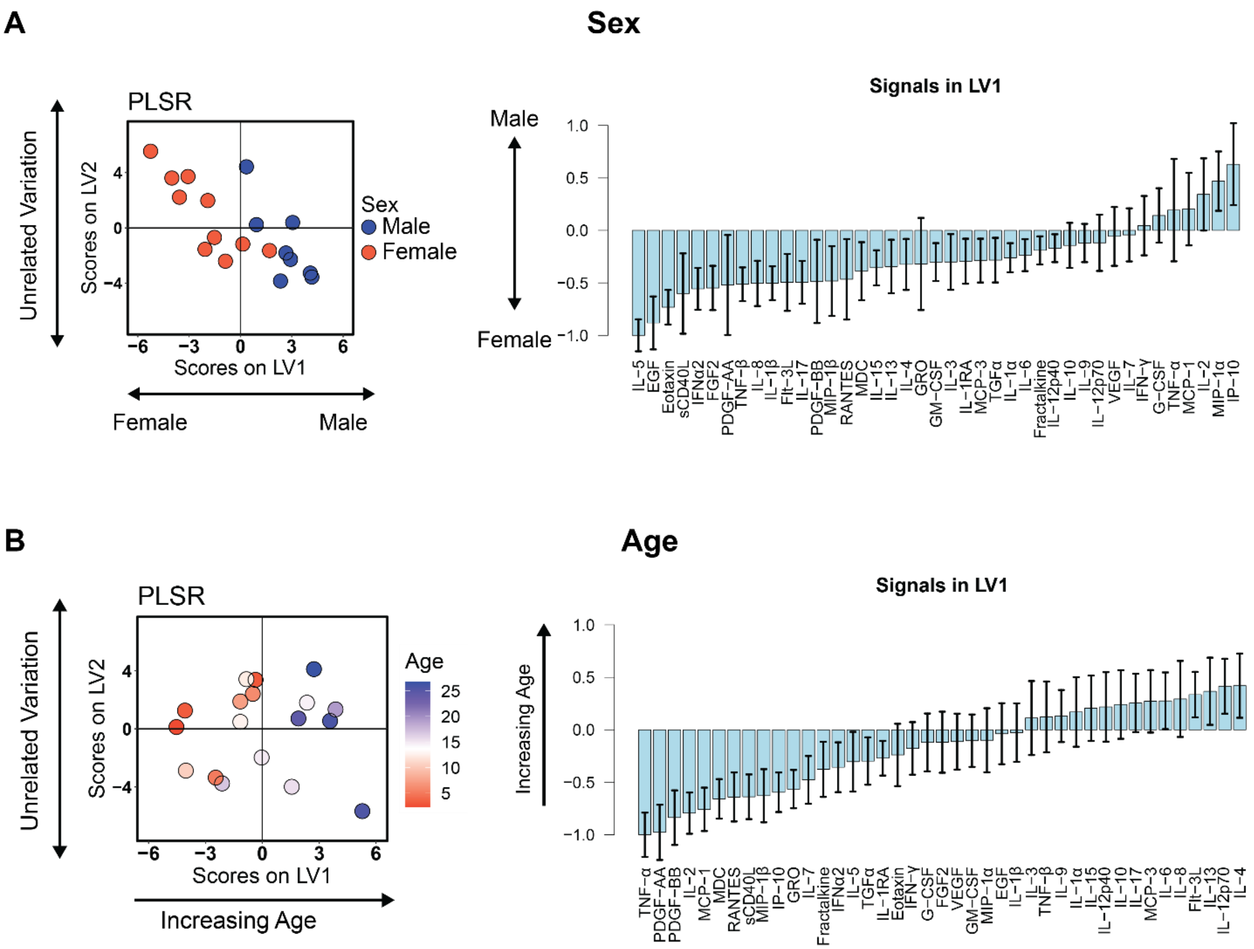

Figure S1: Blood cytokine signatures are related to sex and age in MLIV patients. (**A**) PLSR regression of MLIV patient cytokines against sex revealed a profile of cytokines (LV1) that correlate with females (negative) or males (positive) (mean±SD in a LKOCV with K=3). (**B**) PLSR regression of MLIV patient cytokines against age revealed a profile of cytokines (LV1) that correlate with increasing age (mean±SD in a LKOCV with K=3).

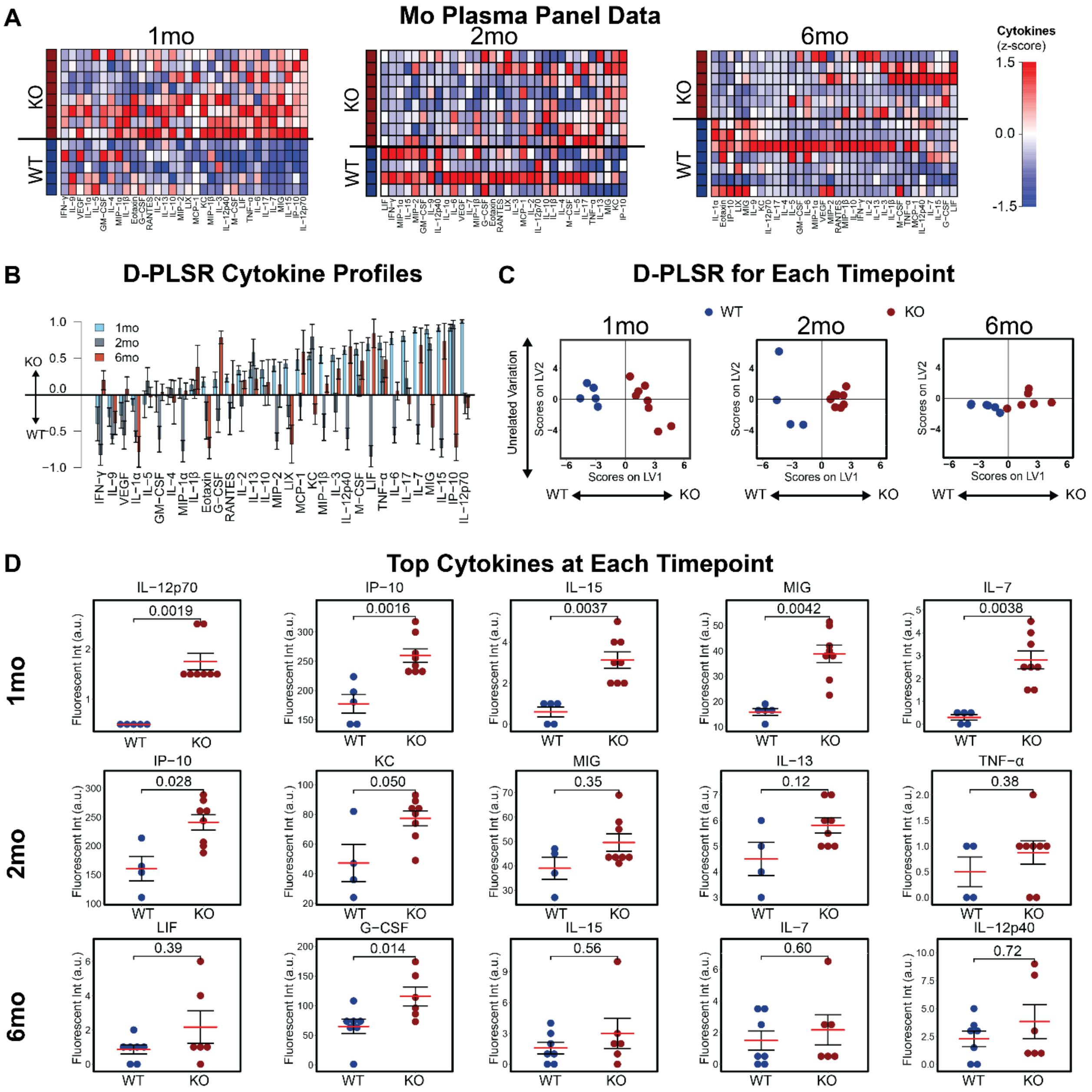

Figure S2: Plasma cytokine signatures distinguish female *Mcoln1*$^{-/-}$ MLIV mice from wild-type controls. (**A**) A panel of 32 cytokines quantified from blood plasma in WT (blue) and *Mcoln1*$^{-/-}$ KO (red) animals at 1 (N=5 WT, 8 KO) , 2 (N=4 WT, 8 KO), and 6 (N=7 WT, 6 KO) months of age (each column is z-scored) (each column is z-scored). (**B**) D-PLSR analysis at each timepoint reveals cytokine signatures associated with WT (negative) or KO (positive) mice (mean±SD in a LKOCV with K=1). (**C**) Scoring each sample from each time point based on its own LV1 profile in (B) separates WT mice to the left and KO mice to the right. (**D**) Univariate analysis of top cytokines from LV1 at 1 (N=5 WT, 8 KO) , 2 (N=4 WT, 8 KO), and 6 (N=7 WT, 6 KO) months of age (mean±SEM, Wilcoxon rank sum test).

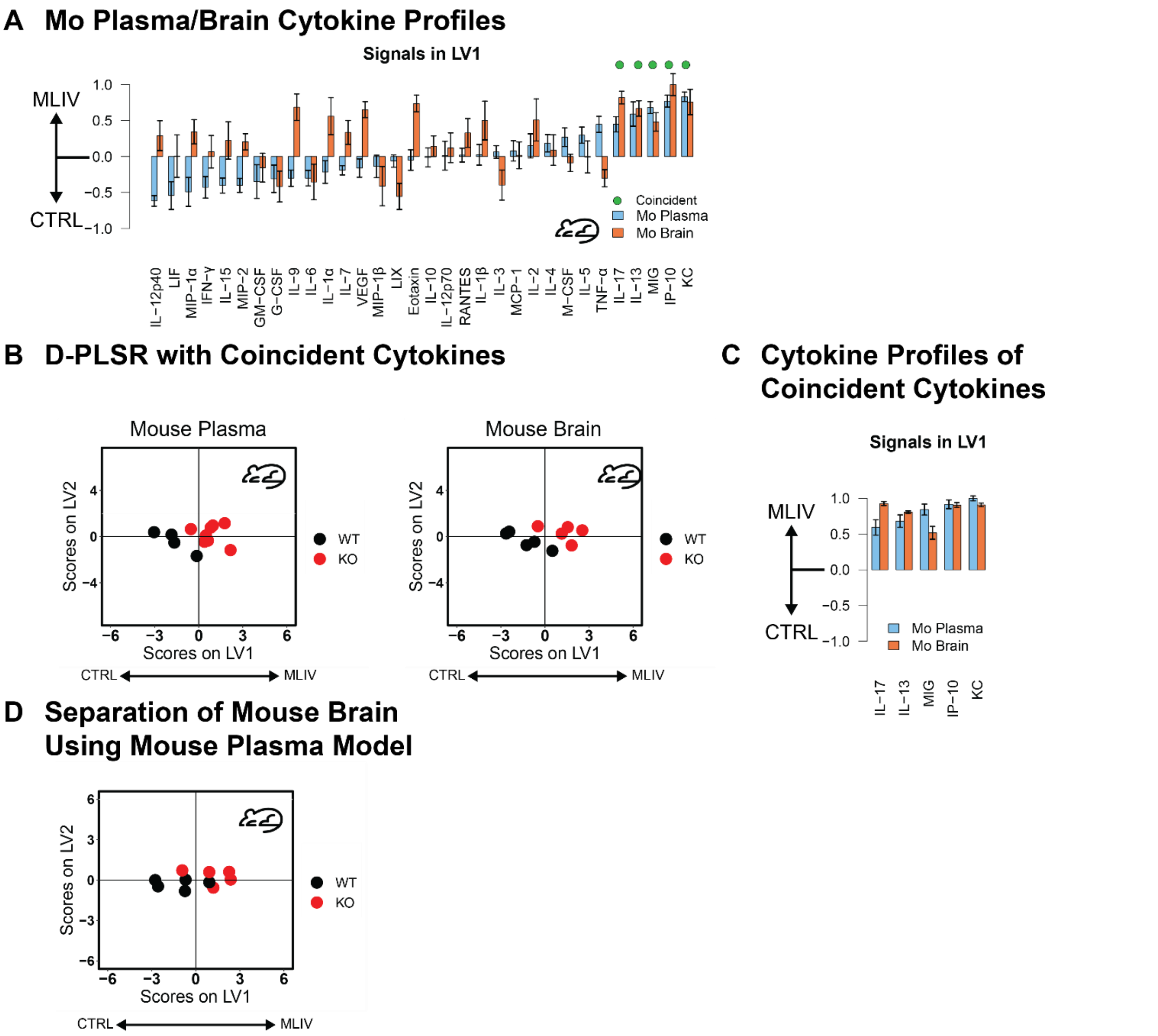

Figure S3: Coincident cytokine signature distinguishes mouse MLIV plasma and mouse brain samples. (**A**) LV1 cytokine profiles distinguishing mouse brain and human plasma generated based on 26 overlapping cytokines in human and mouse datasets. Of these, 5 cytokines were coincident in directionality to separate human plasma and mouse brain MLIV samples from controls. (**B**) D-PLSR analysis with 5 coincident cytokines separated both human plasma and mouse brain samples from controls along LV1, suggesting that this reduced cytokine signature is capable of separating control and MLIV samples from both huma plasma and mouse brain samples. (**C**) Reduced five cytokine LV1 cytokine profiles distinguishing MLIV and control cases for human plasma and mouse brain. (mean±SD in a LKOCV with K=1). (**D**) D-PLSR model generated based on 5 cytokines from human plasma separated wild-type mice to the left and KO mice to the right based on brain cytokine measurements.

Supplementary Tables

Table S1: Statistical analysis of group-wise cytokines differences between CTRL, mild, typical cases. (-) Indicates that fold change (FC) could not be computed because the comparison group had a reading of zero. Statistical testing was conducted via Dunn's Test with Bonferroni correction.

	Typical vs. CTRL		Mild vs. CTRL		Typical vs. Mild	
	p	log2 FC	p	log2 FC	p	log2 FC
IL-12p40	0.004	1.629	1.000	0.155	0.666	1.474
Fractalkine	0.011	0.727	1.000	-0.078	0.216	0.805
FGF2	0.008	1.341	1.000	0.585	0.933	0.756
G-CSF	0.004	0.892	1.000	0.000	0.303	0.892
IL-1β	0.004	1.275	1.000	0.162	0.422	1.113
IL-15	0.003	1.610	1.000	0.061	0.580	1.548
GM-CSF	0.004	1.614	1.000	0.263	0.480	1.350
VEGF	0.000	1.404	0.608	0.819	1.000	0.585
IL-8	0.000	1.541	1.000	0.537	0.489	1.004
IL-6	0.029	1.671	1.000	0.469	1.000	1.202
MIP-1β	0.006	0.716	1.000	0.239	0.860	0.477
IL-1α	0.079	0.922	1.000	-0.485	0.148	1.408
IL-10	0.000	2.050	1.000	0.350	0.530	1.699
IL-13	0.001	2.521	1.000	1.231	0.748	1.290
IL-17	0.012	1.025	1.000	0.646	0.791	0.379
IL-1RA	0.000	1.413	1.000	0.515	0.558	0.898
IL-7	0.008	1.285	1.000	-1.415	0.036	2.700
IL-3	0.028	1.590	1.000	-0.663	0.165	2.253
TNF-β	0.010	1.218	1.000	-0.222	0.208	1.441
Eotaxin	0.009	1.211	1.000	-0.570	0.213	1.781
IL-5	0.017	0.941	1.000	0.138	0.528	0.804
IL-12p70	0.001	1.546	0.810	0.945	1.000	0.602
IFN-γ	0.008	0.954	1.000	0.041	0.558	0.913
MCP-3	0.001	2.312	1.000	1.186	0.306	1.126
IL-4	0.003	2.532	1.000	-	0.064	-
TGF-α	0.001	1.607	1.000	0.206	0.163	1.401
IFN-α2	0.005	0.550	1.000	-0.316	0.053	0.867
MDC	0.000	1.220	1.000	0.366	0.426	0.854
EGF	0.000	3.377	0.487	2.044	0.899	1.332
MIP-1α	0.004	2.074	1.000	0.026	0.379	2.049
MCP-1	0.042	0.766	1.000	-0.668	0.105	1.434
PDGF-BB	0.106	0.985	1.000	-0.495	0.253	1.480
TNF-α	0.001	0.656	0.785	0.311	1.000	0.345
IL-9	0.007	3.283	1.000	-1.430	0.028	4.713
IL-2	0.166	0.558	0.318	-1.000	0.024	1.558
RANTES	0.072	0.751	1.000	0.553	0.826	0.197
PDGF-AA	0.245	0.817	1.000	-0.183	0.511	1.001
IP-10	0.000	2.275	0.871	0.264	0.511	2.011
sCD40L	0.080	1.031	1.000	1.481	1.000	-0.450
Flt-3L	1.000	-0.115	1.000	-0.231	1.000	0.115
GRO	0.035	1.138	1.000	1.692	1.000	-0.554

Table S2: Statistical analysis of group-wise cytokines differences between male WT and KO mice. (-) Indicates that fold change (FC) could not be computed because the comparison group had a reading of zero. Statistical testing was performed using the Wilcoxon rank sum test.

	1mo		2mo		6mo	
**Cytokine**	**p**	**Log2 FC**	**p**	**Log2 FC**	**p**	**Log2FC**
GM-CSF	0.084	1.152	0.606	-4.567	0.014	2.907
MIP-1β	0.023	1.784	1.000	-2.342	0.099	-
MIG	0.054	0.962	0.016	1.535	0.227	0.352
M-CSF	0.203	1.874	1.000	-3.587	0.077	4.587
IP-10	0.114	0.617	0.063	0.994	0.082	0.698
LIF	0.440	0.585	0.118	-2.322	0.768	-2.070
MIP-2	0.209	0.415	0.101	0.678	0.131	2.544
IL-15	0.100	0.946	0.702	0.485	0.014	2.544
IL-12p40	0.584	0.415	0.699	0.485	0.090	3.196
IL-12p70	0.142	1.322	0.770	-5.662	0.461	0.737
G-CSF	0.352	0.555	0.063	0.912	0.052	1.446
RANTES	0.399	0.472	0.375	-1.390	0.298	1.010
MCP-1	0.464	0.415	0.681	-0.644	0.682	0.737
IL-4	0.556	0.263	0.073	-2.074	0.913	-0.070
IL-1β	1.000	0.107	0.416	-0.482	0.191	1.022
TNF-α	0.878	0.074	1.000	-0.750	0.191	1.392
IL-6	0.283	0.366	0.712	-0.597	0.261	1.115
IL-10	0.542	0.415	0.899	-4.144	0.152	2.059
IL-17	0.449	0.214	0.806	-1.029	0.462	0.921
IL-7	1.000	0.232	0.338	1.026	0.042	-
MIP-1β	1.000	0.830	1.000	-4.518	1.000	-
KC	0.914	-0.220	0.111	1.482	0.234	0.581
VEGF	0.324	-0.784	0.266	0.833	1.000	1.032
IL-1α	0.829	-0.134	0.027	2.126	0.027	1.239
Eotaxin	0.762	-0.036	0.413	0.613	0.009	1.996
IL-9	0.591	0.183	0.900	0.534	0.130	2.737
IL-3	0.717	-0.203	0.500	-2.322	1.000	-
IL-13	1.000	-	1.000	-	1.000	-
IFN-γ	1.000	-	0.380	-5.757	0.104	-
IL-2	0.610	-0.457	0.063	0.861	0.851	-0.052
IL-5	0.914	0.165	0.110	0.764	0.141	-1.494
LIX	0.610	0.486	0.016	3.271	0.052	1.442

Table S3: Statistical analysis of group-wise cytokines differences between female WT and KO mice. (-) Indicates that fold change (FC) could not be computed because the comparison group had a reading of zero. Statistical testing was performed using the Wilcoxon rank sum test.

	1mo		2mo		6mo	
**Cytokine**	**p**	**Log2 FC**	**p**	**Log2 FC**	**p**	**Log2 FC**
IL-12p70	0.002	1.807	0.468	-0.415	0.448	-1.462
IP-10	0.002	0.549	0.028	0.584	0.101	-0.385
IL-15	0.004	2.381	0.101	-1.107	0.561	0.933
MIG	0.004	1.297	0.349	0.346	0.252	-0.649
IL-7	0.004	3.229	0.267	-1.341	0.604	0.531
IL-17	0.031	1.109	0.651	0.107	0.716	-2.312
IL-6	0.037	0.538	0.155	-1.541	0.251	-0.226
LIF	0.025	1.239	0.116	-2.737	0.390	1.338
TNF-α	0.019	1.087	0.384	0.807	0.604	0.310
M-CSF	0.066	1.459	1.000	0.000	0.882	0.959
IL-12p40	0.088	1.170	0.188	-0.907	0.718	0.746
MIP-1β	0.120	0.595	0.386	-0.729	0.172	0.162
IL-3	0.110	1.129	0.911	-1.585	0.584	1.338
KC	0.011	0.958	0.050	0.712	0.391	-0.974
MCP-1	0.401	0.985	0.766	-0.415	1.000	0.445
LIX	0.093	1.144	0.214	-0.633	0.295	-2.226
MIP-2	0.089	0.621	0.105	-0.778	1.000	-0.031
IL-13	0.228	0.205	0.116	0.369	0.133	0.373
IL-2	0.181	0.997	0.798	-0.510	0.098	0.200
IL-10	0.214	0.503	0.927	0.000	0.239	-0.643
RANTES	0.233	0.788	0.731	-0.553	0.772	-0.948
G-CSF	1.000	0.169	0.808	-0.471	0.014	0.834
Eotaxin	0.354	0.622	0.683	-1.089	0.073	-2.118
IL-1β	0.571	0.474	0.766	0.170	1.000	-0.041
MIP-1α	0.453	0.946	0.147	-4.170	0.774	-0.328
GM-CSF	0.357	0.222	0.531	-1.138	0.089	-0.418
IL-4	0.641	0.206	1.000	0.000	0.941	-3.700
IL-5	0.826	-0.107	0.496	0.182	0.668	-0.327
IL-1α	1.000	0.136	0.125	-2.643	0.234	-1.652
VEGF	0.811	-0.093	0.319	-0.924	1.000	0.191
IL-9	1.000	-0.290	0.144	-1.666	0.279	-1.788
IFN-γ	0.695	-0.919	0.050	-	0.329	0.729
